# Transcarotid TAVR for Severe Bicuspid Aortic Stenosis With Virtually Atretic Coarctation of the Thoracic Aorta

**DOI:** 10.1016/j.jscai.2024.101940

**Published:** 2024-04-11

**Authors:** Robert M. Tungate, Majed Chane, Jack Sun, Anil K. Tiwari, Deniz Urgun, Pranav M. Patel, Antonio H. Frangieh

**Affiliations:** a Division of Cardiology, Department of Medicine, University of California Irvine Medical Center, Orange, California; b Fountain Valley Regional Hospital, Huntington Beach, California; c Division of Cardiothoracic Surgery, Department of Surgery, University of California Irvine Medical Center, Orange, California; d Department of Anesthesiology & Preoperative Care, University of California Irvine Medical Center, Orange, California; e Department of Radiological Sciences, University of California Irvine Medical Center, Orange, California

**Keywords:** bicuspid aortic valve, coarctation of the aorta, congenital heart disease, transcarotid access, transcatheter aortic valve replacement

## Abstract

Transcatheter aortic valve replacement by alternate access sites allows for treatment of patients with unfavorable anatomy for a transfemoral approach. To our knowledge, we present the first reported case of successful transcatheter aortic valve replacement via the transcarotid approach in a 65-year-old man with a symptomatic severe bicuspid aortic valve stenosis and virtually atretic coarctation of the thoracic aorta.

## Introduction

Transcarotid access has been shown to be a safe, effective alternative strategy for transcatheter aortic valve replacement (TAVR) for patients in whom a transfemoral approach is unfavorable.^1^^,^^2^ To our knowledge, we present the first reported case of successful transcarotid bicuspid aortic valve (BAV) TAVR where transfemoral access was not feasible due to congenital, virtual coarctation of the thoracic aorta.

## Case Presentation

A 65-year-old man with congenital BAV and hypertension was referred to our center for symptomatic severe aortic valve stenosis (AS) in the context of total occlusion of the aorta distal to the left subclavian artery. The patient reported worsening exertional shortness of breath for several months. Physical examination revealed a well-developed man with a systolic murmur. Blood pressure and pulse timing and amplitude were symmetric in all extremities.

## Investigations

**Figure 1:**
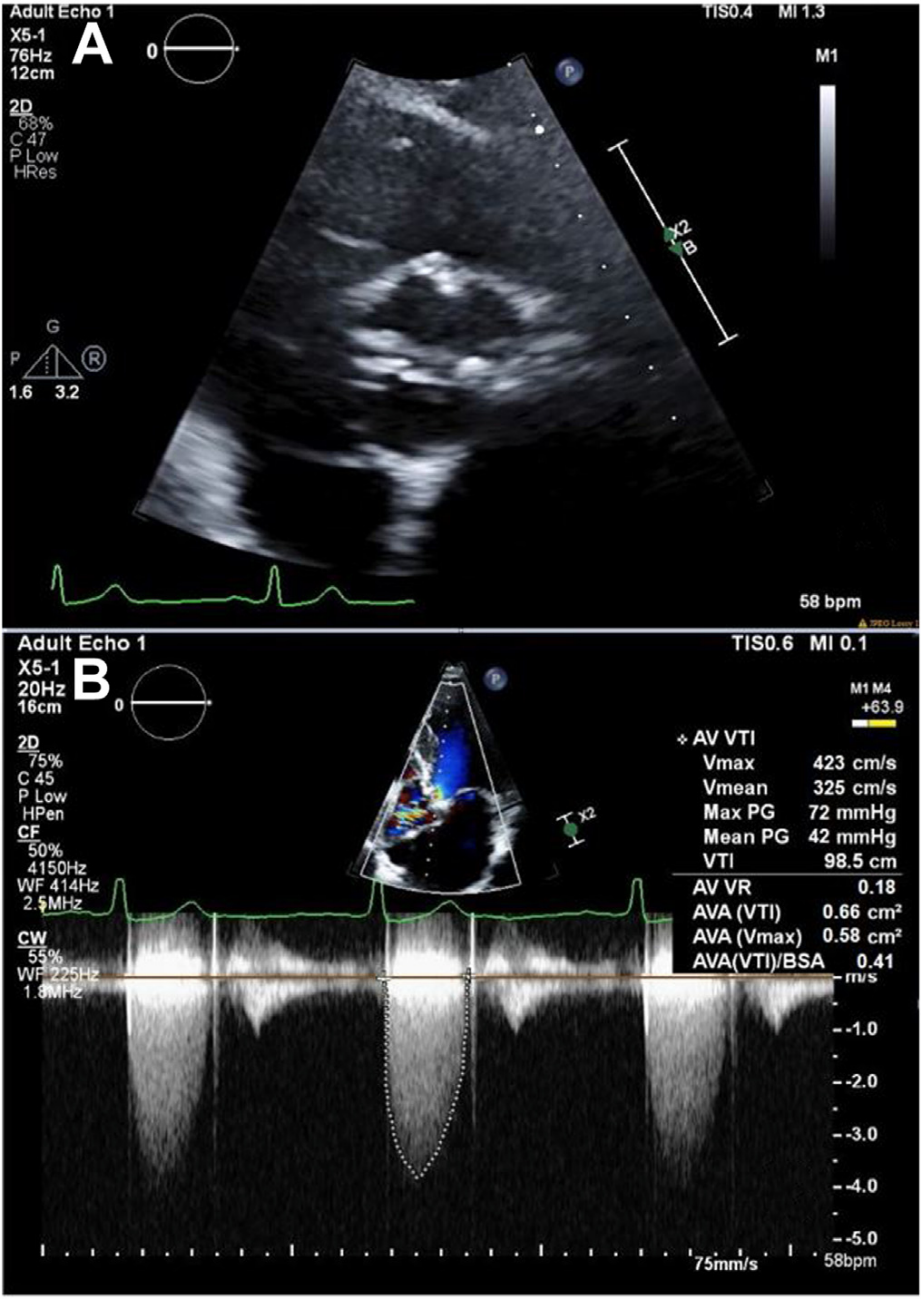
**Transthoracic echocardiogramof the aortic valve and continuous wave transaortic valve doppler.** (**A**) A short-axis TTE view of the bicuspid aortic valve. (**B**) TTE with Doppler analysis reveals an aortic valve peak velocity of 4.23 m/s and a peak transaortic PG of 72 mm Hg, mean transaortic PG of 42 mm Hg, and a calculated aortic valve area (AVA) of 0.66 cm^2^, corresponding to an AVA-to-body mass index of 0.41 cm^2^/m^2^.

**Figure 2:**
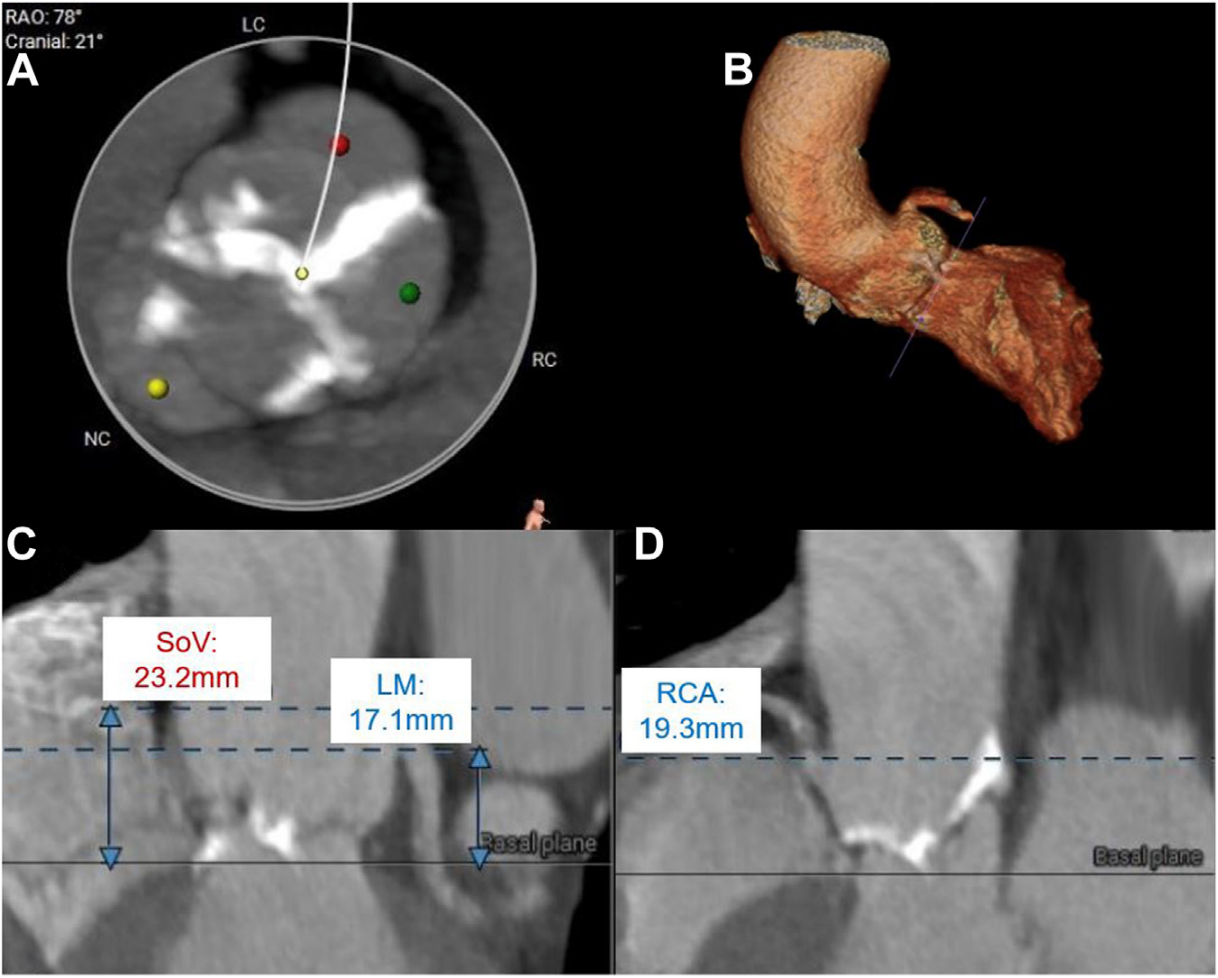
**Cardiac CT with contrast.** (**A**) Cardiac CT reveals a calcified fusion of the left and right coronary cusps. The aortic valve annulus is measured as 20 × 27 mm with an area of 419 mm^2^ and a circumference of 47 mm with an aortic valve calcium score of 2798. (**B**) Three-dimensional reconstruction of the sinuses of Valsalva. The origin of the left coronary artery from the sinus of Valsalva is seen. (**C**) Left coronary artery to annulus height of 17 mm. Sinus of Valsalva height is measured as 23 mm with a width of 25 × 27 × 33 mm. (**D**) Right coronary artery to annulus height of 19 mm. CT, computed tomography.

**Figure 3:**
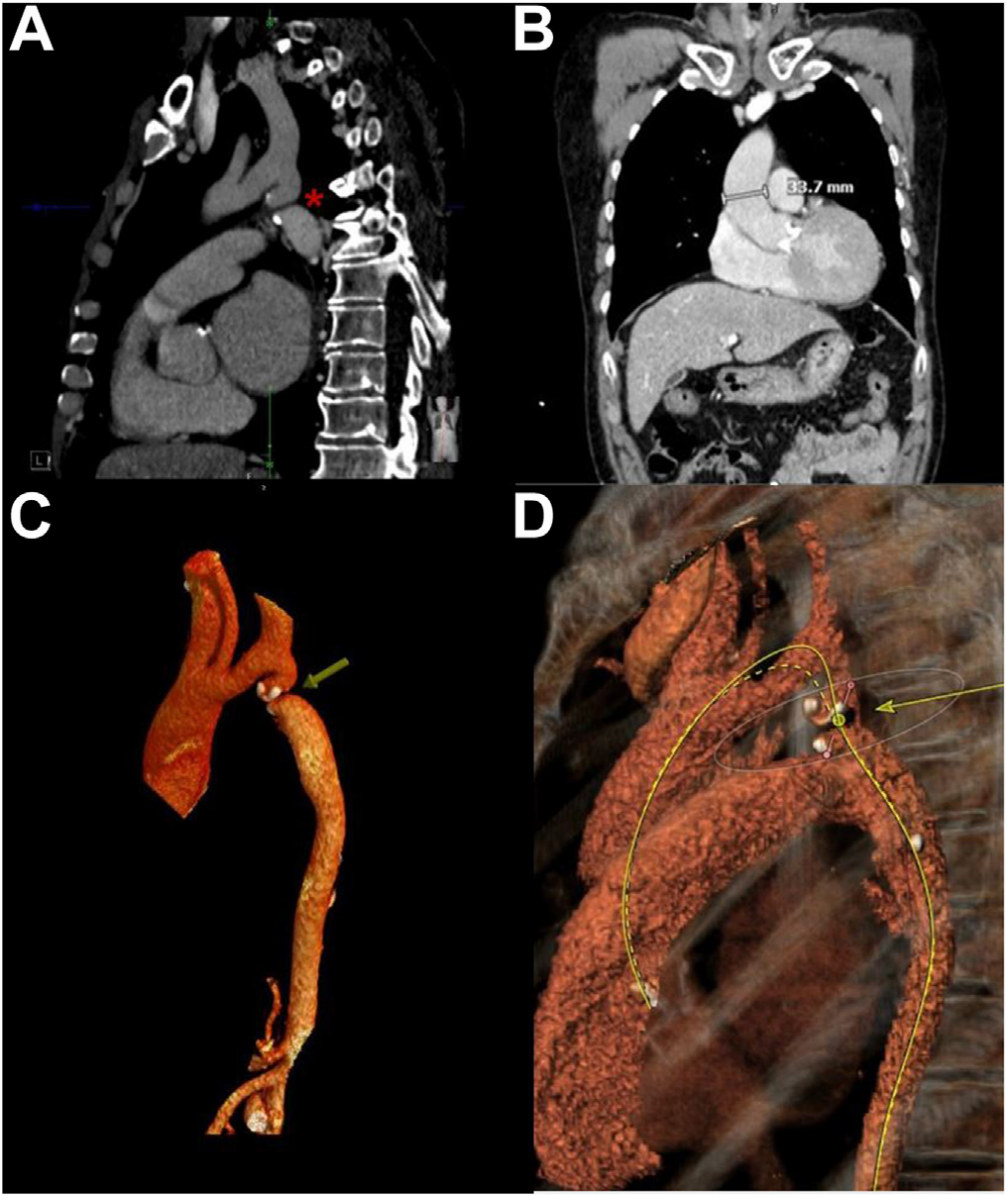
**CTangiography of the thoracic and abdominal aorta.** (**A**) The patient’s coarctation of the descending aorta with virtual atresia (marked with a red asterisk) as shown in the coronal plane by CT scan. The atretic segment rests 14.7 mm below the ostium of the left subclavian artery. Despite virtual atresia of the aorta, forward flow through the coarctation is observed. The subclavian artery and branches are dilated, providing collateral flow to structures distal to the coarctation. (**B**) The ascending aortic diameter is 33.7 mm as measured by coronal plane CT. The bicuspid aortic valve is heavily calcified. (**C**) The patient’s ascending aorta is atretic due to a calcified coarctation, rendering transfemoral transcatheter aortic valve replacement access infeasible. (**D**) Reconstructed CT of the thoracic aorta reveals that the aortic coarctation obstructs the approach to the aortic valve from the descending aorta. As a result, the modeled approach path is not feasible. CT, computed tomography.

**Figure 4:**
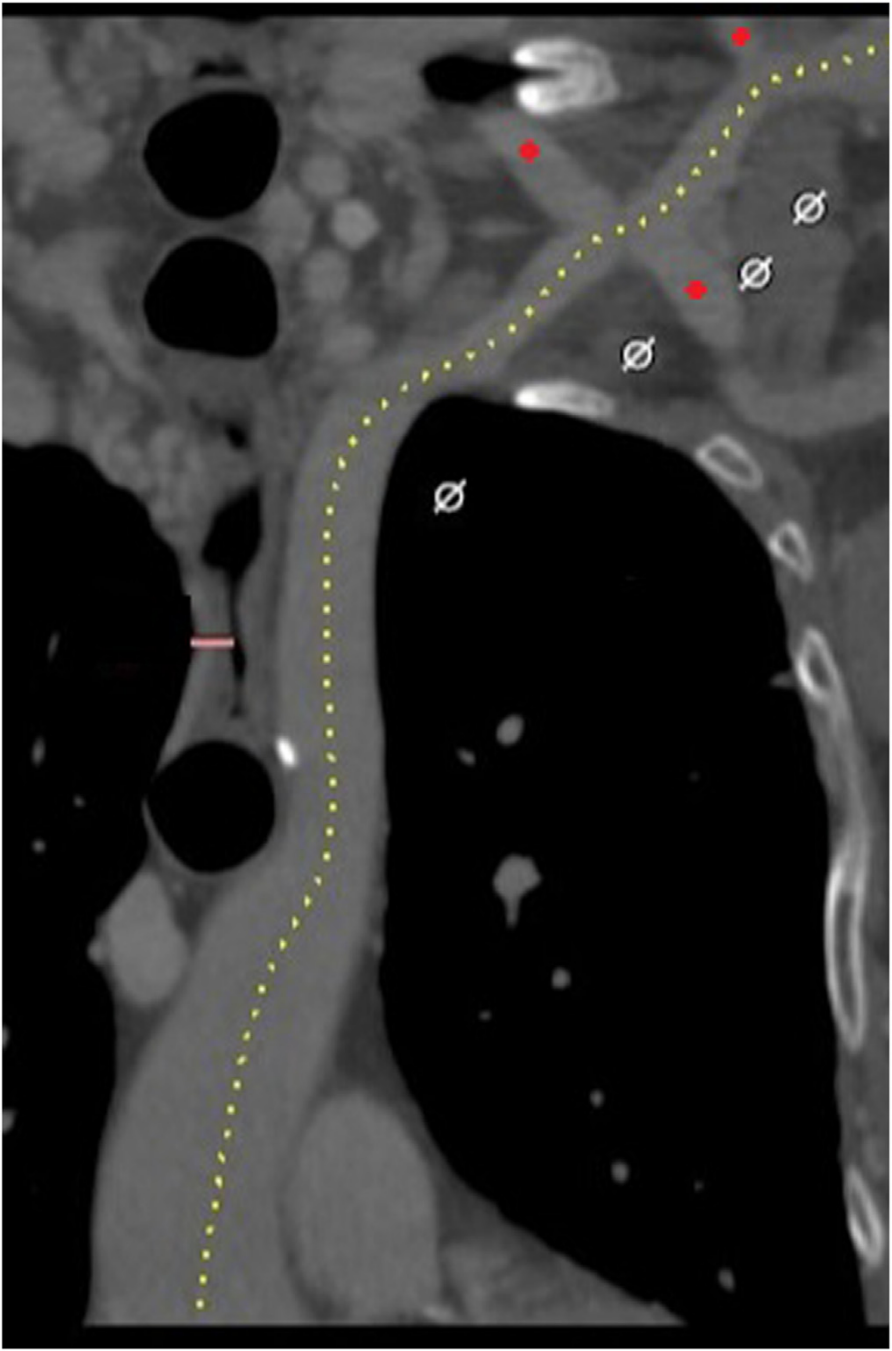
**A CTangiogram of the left subclavian artery.** Large collateral branches that bypass the coarcted aorta originate from the left subclavian artery and are marked with a red button. A left carotid, axillary artery, or subclavian artery for transcatheter aortic valve replacement was not selected in this case to not disrupt these collateral branches. CT, computed tomography.

Electrocardiogram showed sinus rhythm with left ventricular hypertrophy and nonspecific T-wave inversions. Transthoracic echocardiogram (TTE) revealed a normal left ventricular ejection fraction of 58%, severe diastolic dysfunction, and a severely stenotic, calcified aortic valve (AV). The maximum velocity across the valve was 4.23 m/s and the mean pressure gradient (PG) was 42 mm Hg. AV area by the continuity equation was 0.58 cm^2^ (Figure 1). Coronary angiography showed nonobstructive coronary artery disease with nonhemodynamically significant 50% left anterior descending stenosis. Gated, contrast-enhanced computed tomography (CT) revealed a Sievers type I BAV with calcified raphe, fusion of the right and left coronary cusps, and calcium protruding into the left ventricular outflow tract. Measurements included an AV annulus of 20 × 27 mm with an area of 419 mm^2^ (Figure 2). The AV calcium score was 2798, consistent with severe AS. Thoracic angiography revealed a focal occlusion of the proximal thoracic aorta with a thin membrane approximately 1.4 cm below the ostium of the left subclavian artery with dilated internal mammary, bronchial, and intercostal arteries (Figures 3 and 4). Despite severe coarctation (COA), some forward flow to the distal aorta was observed on the CT scan.

## Management

After a multidisciplinary heart team discussion, the patient was considered an acceptable surgical risk for surgical AV replacement because of the nonfeasibility of transfemoral TAVR, his anatomy, and his youth. After a thorough conversation, the patient declined surgery and elected to proceed with TAVR. The team prioritized extrathoracic percutaneous access for TAVR. The transcarotid approach via the right common carotid artery (CCA) was selected for multiple reasons detailed in the discussion below.

**Figure 5:**
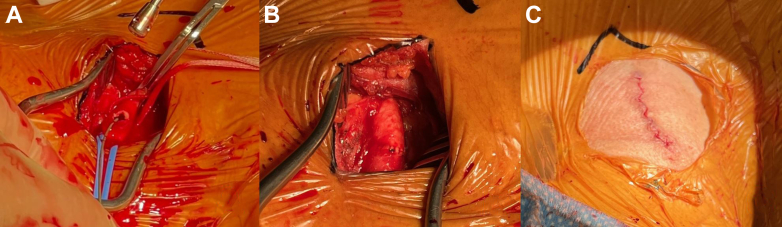
**Carotidarterycutdown.** (**A**) The dissected carotid artery during cannulation. (**B**) The right common carotid artery is closed with a vascular closure device. (**C**) The sutured carotid incision and cut-down site.

The procedure was performed under general anesthesia. The right CCA was dissected out^3^ (Figure 5). A transvenous pacing wire was inserted. From the left radial artery, a root angiogram was performed to confirm coaxial fluoroscopy angles.

**Figure 6:**
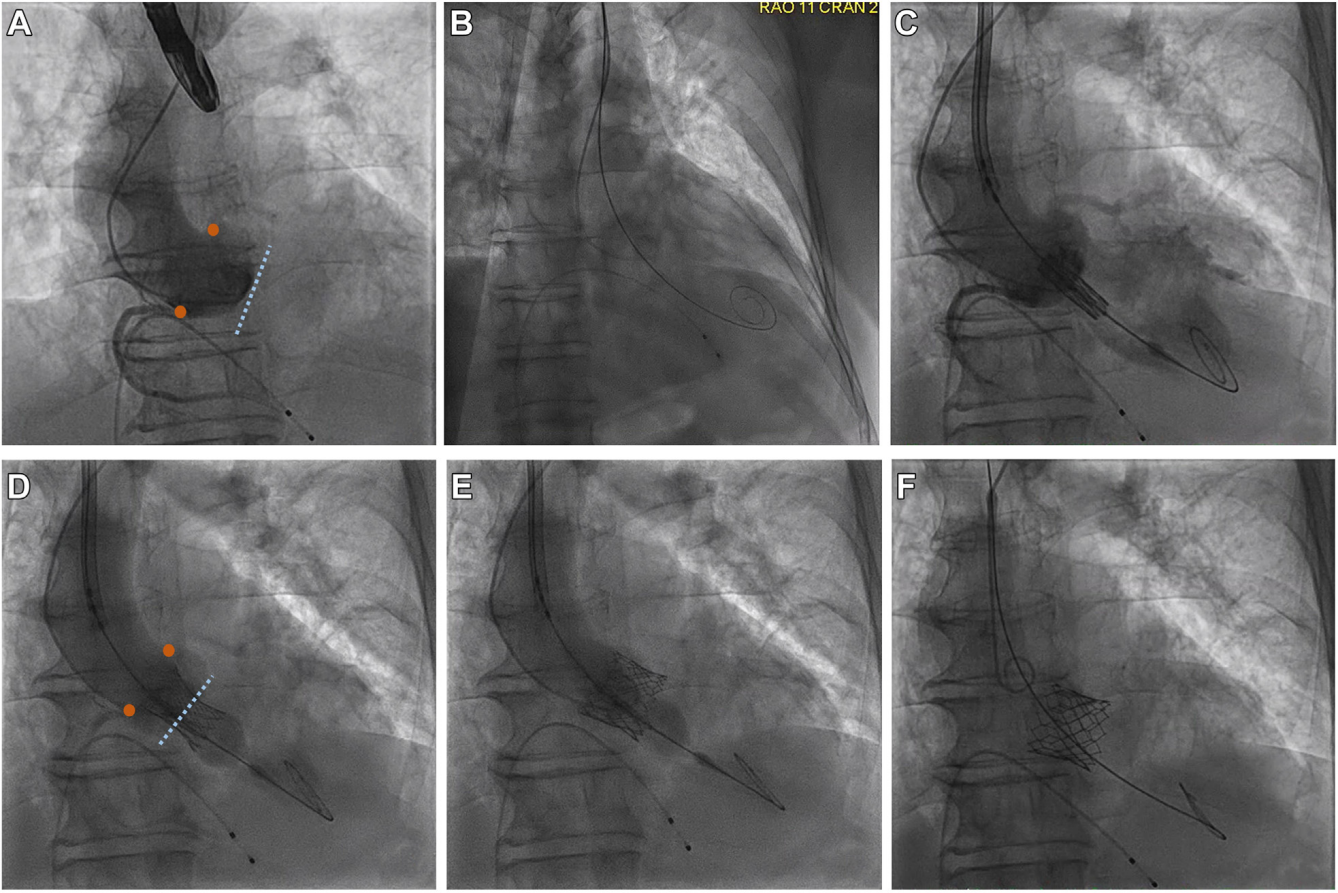
**Coaxial deployment of the transcatheter aortic valve replacement with an angiogram, in progression from A to F.** Coronary ostia are indicated by the orange markers. The coaxial plane of the aortic valve annulus is marked by the dotted blue line.

Through the transcarotid access, the AV was crossed with a stiff, preshaped wire. The proximal CCA was clamped proximally. The 6F sheath was removed and the artery was transected along the guide wire. A 14F E-Sheath was then advanced over the guide wire and into the aortic arch. A balloon aortic valvuloplasty interrogation was performed for valve sizing. Then, a 26 mm SAPIEN 3 Ultra RESILIA bioprosthetic transcatheter heart valve (Edwards Lifesciences) was positioned using angiography. It was successfully deployed under rapid pacing (Figure 6). Invasive pressure gradient showed excellent hemodynamics with no peak-to-peak PG. A control aortic angiogram demonstrated excellent positioning of the valve with no paravalvular leak. Postprocedural TTE measured the mean trans-AV PG as 3 mm Hg. The E-Sheath was removed and the carotid artery was closed with a vascular suture delivery system (Figure 5).

The patient’s postoperative course was uncomplicated. On hospital day 3, a transthoracic echocardiogram revealed an unchanged normal left ventricular ejection fraction. The bioprosthetic AV was without perivalvular leak. The peak aortic velocity was 1.76 m/s and the mean trans-AV PG was 6.6 mm Hg.

The patient was discharged on hospital day 3. He was assessed at follow-up visits 10 days and 30 days after the TAVR procedure. At these visits, he reported feeling well. His exercise capacity was improved. At his 30-day follow-up visit, TTE demonstrated a well-functioning bioprosthetic AV with an unchanged mean transaortic PG and no perivalvular leak. Two months after TAVR, he was walking 30 minutes daily without dyspnea.

## Discussion

BAV has an incidence of 0.5% to 2% in the population and is considered the most common congenital cardiac anomaly.^4^ BAV may be associated with COA, and up to 50% of all patients with coarcted aorta are estimated to have BAV.^4^ BAV is known to be prone to senile calcific stenosis.^4^ Given the prevalence of BAV senile calcific AS and COA, options for interventional management of this population are of great interest in both early- and late-presenting cases. The syndrome should be suspected in patients with a left upper sternal ejection murmur, refractory hypertension, and a differential in blood pressure and pulse strength between the upper and lower extremities.^4^ If left untreated, in severe cases, LV dysfunction and hypertensive organ injury can occur.

This patient’s coarcted aorta was virtually atretic (Figure 3). In this case, unfortunately, the COA was not diagnosed until adulthood. Systemic collateral revascularization via the right-sided innominate, subclavian, mammary, and bronchial arteries had bypassed the atretic aortic segment to help the patient compensate. Blood pressure and pulse were nearly symmetric, attributed to this robust collateral flow. This may explain why the patient’s COA had not been diagnosed earlier in life. Recanalization of the COA with a covered stent was considered to improve LV function, aortic flow, and secondary hypertension. However, the heavy calcifications at the coarct site posed a high risk of complications with transcatheter intervention. As the patient was well-compensated prior to developing severe AS, no intervention on the COA was performed.

In this patient, access from the groin, including standard transfemoral or transcaval approaches, was not feasible. Alternative approaches were considered. When compared with transapical or transaortic approaches, multicenter studies of transcarotid TAVR have demonstrated shorter hospitalizations, fewer strokes, and trend toward lower mortality.^2^^,^^5^ Meta-analyses do not show a difference in stroke or mortality between transfemoral and transcarotid TAVR approaches despite a generally elevated risk profile in patients selected for transcarotid cases.^1^ For this patient, arterial supply distal to the COA depended on collateralizing branches of the left subclavian and suprathoracic arteries (Figure 4). Right carotid access, therefore, was preferred to the left axillary artery or subclavian access to minimize any potential vascular complications to these vital collateralizing thoracic arteries. Supporting data also suggest a lower stroke risk with transcarotid compared with subclavian access.^5^

Patients with BAV were excluded from TAVR landmark randomized clinical trials, but the safety and efficacy of TAVR with balloon-expandable valves in patients with BAV is established,^6^ and TAVR may be considered as an alternative to surgical AV replacement in select patients (an American Heart Association/American College of Cardiology class 2B recommendation).^7^ In TAVR of BAV, valve positioning and expansion are affected by BAV’s elliptical shape, larger size, and eccentric calcification.^8^ Calcified raphe and excess leaflet calcification, both of which were present in this case, are risk factors for early mortality or paravalvular leak.^9^ CT-based measurements may underestimate annulus area as they assume shapes more typical of trileaflet valves.^8^ Coaxial alignment of the catheter and the valve during crossing of the valve and deployment are imperative to minimize the risk of complications.^8^ This is best achieved with predefinition of valve anatomy with CT scan.^10^ The effects of distal stenosis due to COA on the risk of relatively early degeneration of bioprosthetic AV are not well-characterized. Careful surveillance for evidence of prosthetic valve degeneration or regurgitation is indicated.

## Conclusion

To our knowledge, we present the first reported case of successful transcarotid TAVR for a 65-year-old man with the syndrome of severely stenotic, calcified BAV and coarctation of the thoracic aorta with excellent results. Transcarotid TAVR is a feasible alternative approach for this patient population.
